# Prevalence, genetic variants and clinical implications of G-6-PD deficiency in Burkina Faso: a systematic review

**DOI:** 10.1186/s12881-017-0496-2

**Published:** 2017-11-23

**Authors:** Abdoul Karim Ouattara, Pouiré Yameogo, Lassina Traore, Birama Diarra, Maléki Assih, Tegwindé Rébéca Compaore, Dorcas Obiri-yeboah, Serge Théophile Soubeiga, Florencia Wendkuuni Djigma, Jacques Simpore

**Affiliations:** 1 Pietro Annigoni Biomolecular Research Center (CERBA), 01 PO BOX 364, Ouagadougou 01, Burkina Faso; 2 Laboratory of Molecular Biology and Molecular Genetics (LABIOGENE) UFR/SVT, University Ouaga I Prof Joseph KI-ZERBO, Ouagadougou 03, BP 7021 Burkina Faso; 3 Department of Microbiology and Immunology, University of Cape Coast, Cape Coast, Ghana

**Keywords:** G-6-PD deficiency, Polymorphism, Haplotype, Malaria, Burkina Faso

## Abstract

**Background:**

It is now well-known that some antimalarials such as primaquine may induce severe hemolytic anemia in people with G-6-PD deficiency. Antimalarial drug prescriptions must, therefore take into account the patient’s G-6-PD status in malaria endemic areas such as Burkina Faso, where the prevalence of this genetic abnormality is relatively high. Although great clinical heterogeneity is observed depending on the molecular nature of the deficiency and the residual enzyme activity in the red blood cell, there is very poor data on the prevalence of G-6-PD deficiency and the distribution of involved genetic variants in Burkina Faso. In this systematic review, we present a synthesis of the various studies carried out on the G-6-PD deficiency in Burkina Faso in order to determine its prevalence, probable distribution of the genetic variants involved and their clinical implications for a national systematic screening policy among the groups most vulnerable to malaria.

**Methods:**

A systematic review was carried out to analyze available published data on the prevalence, phenotypes and mutations responsible for G-6-PD deficiency in Burkina Faso. The key words used were “G-6-PD deficiency AND Burkina Faso” or “Déficit en G-6-PD AND Burkina Faso” in French. To identify the relevant articles, two independent reviewers reviewed the titles, abstracts and the full text of the selected papers.

**Results:**

An average prevalence of 16.6% (183/1100; CI 95%: 0.145–0.190) and 6.5% (69/1066; CI 95%: 0.051–0.081) of G-6-PD deficiency was found respectively in men and women in this systematic review. Although the predominance (99.8% of G-6-PD deficient cases) of 202A/376G G-6-PD A- variant, the Santamaria and Betica Selma variants were identified in Burkina Faso. Independently of the method used, the enzymatic deficiency was significantly higher in males (2.5–20.5%) compared to females (3.3–12.3%).

**Conclusion:**

This systematic review suggests that despite the ubiquity of the 202A/376G G-6-PD A- variant in Burkina Faso, it will be necessary to consider the Santamaria and Betica Selma variants although their frequencies remain to be specified. A systematic screening of the G-6-PD deficiency is also needed to prevent the occurrence of iatrogenic hemolytic accidents.

## Résumé

**Contexte:**

Il est. actuellement bien connu que certains antipaludiques comme la primaquine, peuvent induire des crises d’anémie hémolytique graves chez les personnes présentant un déficit en G-6-PD. Les prescriptions de médicaments antipaludiques doivent donc tenir compte du statut G-6-PD du patient dans les zones d’endémie du paludisme comme le Burkina Faso où la prévalence de cette anomalie génétique est. relativement élevée. En dépit d’une grande hétérogénéité clinique observée selon la nature moléculaire du déficit et l’activité résiduelle de l’enzyme dans le globule rouge, il existe très peu de données sur la prévalence du déficit en G-6-PD et la distribution des variants génétiques en cause au Burkina Faso. Dans cette revue de la littérature nous présenterons la synthèse des différents travaux réalisés sur le déficit en G-6-PD au Burkina Faso afin de déterminer sa prévalence, la distribution probable des variants génétiques en cause et leurs implications cliniques en vue d’une politique nationale de dépistage systématique au sein des groupes les plus vulnérables au paludisme.

**Méthodes:**

Une revue systématique a été réalisée pour analyser les données publiées disponibles sur la prévalence, les phénotypes et les mutations du déficit en G-6-PD au Burkina Faso Les mots clés utilisés étaient « G6PD deficiency AND Burkina Faso » en anglais ou « Déficit en G6PD AND Burkina Faso en français ». Pour identifier les articles pertinents, deux examinateurs indépendants ont examiné les titres, les résumés et le texte intégral des articles retenus.

**Résultats:**

Une prévalence moyenne de 16,6% (183/1100; IC 95%: 0,145–0,190) et 6,5% (69/1066; IC 95%: 0,051–0,081) du déficit en G-6-PD a été observée respectivement chez les hommes et les femmes dans cette revue systématique. Malgré la prédominance (99,8% des cas de déficients en G-6-PD) du variant G-6-PD A- 202A/376G, les variants Santamaria et Betica Selma ont été identifiées au Burkina Faso.

Indépendamment de la méthode utilisée, la prévalence du déficit enzymatique était significativement plus élevée chez les hommes (2,5–20,5%) comparativement aux femmes (3,3–12,3%).

**Conclusion:**

Cette revue systématique suggère qu’en dépit de l’ubiquité du variant G-6-PD A- 202A/376G au Burkina Faso, il est. nécessaire de prendre en compte les variants Santamaria et Betica Selma, bien que leurs fréquences restent à préciser. Un dépistage systématique de la déficience en G-6-PD est. également nécessaire pour prévenir la survenue d’accidents hémolytiques iatrogènes notamment chez les populations les plus vulnérables au paludisme.

## Background

Glucose-6-phosphate dehydrogenase (G-6-PD) is a key enzyme (EC 1.1.1.49) of the pentose phosphate pathway for the production of Nicotinamide Adenine Dinucleotide Phosphate (NADPH) and ribose-5-phosphate [1–3]. It plays a very important role in oxidative stress control in red blood cells without nucleus and mitochondria. A dysfunction of this enzyme, therefore makes the erythrocyte vulnerable to oxidative damage [4]. The clinical presentation of G-6-PD deficiency, however, depends on the level of enzyme deficiency and the intensity of oxidative stress within the erythrocytes [5]. G-6-PD deficiency is a human genetic abnormality with high frequency in malaria endemic areas. G-6-PD gene is highly polymorphic with many allelic variants responsible for different levels of enzymatic deficiency and causing various clinical manifestations [6–8]. The different variants responsible for the G-6-PD deficiency were grouped into five (5) classes according to the level of the erythrocyte enzyme activity (≤ 1 to 150%) and the importance of the clinical manifestations [9–11]. The most common deficient haplotype or G-6-PD A- in sub-Saharan Africa has two mutations in cis. These are the G202A (rs1050828) and A376G (rs1050829) mutations with a high linkage disequilibrium [8]. Other alleles responsible for the G-6-PD deficiency with frequencies that are over 1% have also been reported in West Africa.

The latter are represented respectively by T968C (rs76723693) and A542T (rs5030872) substitutions [8, 12–14]. G-6-PD deficiency is the most common enzymopathy affecting about 7% of the world population [15]. Former studies have suggested that the geographic distribution of G-6-PD deficiency, which is highly correlated with the distribution of current or past malaria endemicity, is not a matter of chance but reflects the only significant selective advantage conferred to carriers of deficient alleles: a resistance against malaria progression to severe forms [16–19]. Analyses of association between the different G-6-PD genotypes and malaria showed that high levels of G-6-PD deficiency are associated with a decreased risk of cerebral malaria and an increased risk of severe malarial anemia [8, 20].

In Burkina Faso, malaria is highly endemic with an increase in transmission during the rainy season [21]. During this season, it is estimated that more than half of all cases of fever are attributable to malaria [22]. In 2013, malaria remained the leading cause of consultations (46.5%), hospitalizations (61.5%) and deaths (30.5%) in health facilities in Burkina Faso [23]. Under the malaria pressure, the country has a relatively high frequency of G-6-PD deficiency. A particular attention should, therefore be paid to G-6-PD deficiency, which is an X-linked genetic disorder with variable clinical expressions in heterozygous women, which may present serious problems, particularly during malaria treatment [11, 24, 25]. The use of certain antimalarials such as primaquine, consumption of certain foods (fava beans), and a variety of infections (hepatitis, typhoid fever, malaria) induce hemolytic anemia in G-6-PD deficient individuals with various intensity and severity, sometimes requiring emergency blood transfusions [3, 26]. The country should, therefore be informed about the actual prevalence of this enzymatic disorder at the national level as well as the distribution of the genetic variants involved and their clinical implications.

This will allow for safe and appropriate national decisions on the use of potentially dangerous drugs for individuals with G-6-PD deficiency. The aim of this systematic review is to assess the prevalence of G-6-PD deficiency in Burkina Faso and the distribution of the genetic variants involved and their clinical implications for a national systematic screening particularly among the groups most vulnerable to malaria; children under five and pregnant women.

## Methods

**Fig. 1:**
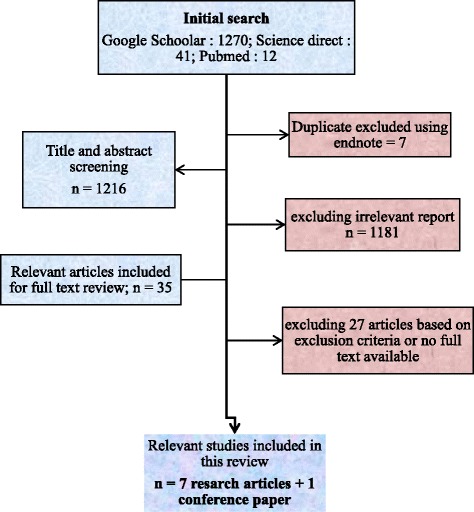
Flow diagram showing the method for the papers selection. The database search according to the search strategy described in the methodology section was clean up to exclude duplicates. Titles and abstract were initially screened to include all relevant studies describing the prevalence and/or genetic variants of the G-6-PD deficiency in Burkina Faso. Review articles, articles without abstract or without full text as well as those that did not meet the inclusion criteria were then excluded during the full-text review. Seven (7) research relevant articles and one (1) conference paper were finally selected for this review of the literature

A systematic review was carried out to analyze available published data on the prevalence, phenotypes and mutations responsible for G-6-PD deficiency in Burkina Faso. Potentially relevant articles in English or French were searched for in PubMed, Google Scholar and Science Direct for a full-text review. The key words used were “G-6-PD deficiency AND Burkina Faso” or “Déficit en G-6-PD AND Burkina Faso” in French. Additional articles were obtained through the follow-up of quotations from journals/opinion articles and original documents. The relevant papers search strategy is presented in Fig. 1. To identify the relevant articles, two independent reviewers reviewed the titles, abstracts and the full text of the selected papers. Prevalences were calculated by plotting the number of people with G-6-PD deficiency in the different studies on the total number of people screened. Confidence Intervals were calculated using the R software version 3.3.3. The haplotypic frequencies of G-6-PD B, A and A- variants were calculated from data from three studies [14, 27, 28]. The populations of these three studies were conform to Hardy-Weinberg equilibrium and these data were used for Inverse Distance weighted (IDW) interpolation of G-6-PD deficiency allele frequency in Burkina Faso using QGIS 2.18.14 software.

## Results

### Prevalence of G-6-PD deficiency in Burkina Faso

The prevalence of G-6-PD deficiency observed in the various studies is presented in Table 1. In all selected studies for this systematic review, an average prevalence of 16.6% (183/1100; CI 95%: 0.145–0.190) and 6.5% (69/1066; CI 95%: 0.051–0.081) of G-6-PD deficiency was found respectively in men and women.

**Table 1:** Prevalence of G-6-PD deficiency in Burkina Faso according to several authors

Characteristics						G-6-PD deficiency	
Sample	Method	Origin	Author	Year	Malaria	Male	Female
498	PCR/RFLP	Oubritenga	Modiano et al.	2001	asymptomatic	^a^16.7%	
222	Enzymatic test	Nouna	Meissner et al.	2005	symptomatic	15,1%	7,0%
750	Enzymatic test	Nouna	Coulibaly et al.	2005	asymptomatic	16.9%	7.6%
342	Enzymatic test	Kadiogo	Simporé et al.	2007	No	20.5%	12.3%
357	PCR/RFLP	Ouagadougou	Carter et al.	2011	symptomatic	15.4%	3.3%
232	Enzymatic test	Banfora	Badoun et al.	2014	symptomatic	4.74%	2.15%
200	Real Time PCR	Koubri	Ouattara et al.	2014	asymptomatic	14.3%	6.0%
182	Classic PCR	Ouagadougou	Ouattara et al.	2016	symptomatic	15.2%	4.4%

^a^No information about prevalence according to gender

According to the methodology used, the prevalence of the deficiency ranged from 15.1 to 20.5% with an average of 17.3% (130/750; CI 95%: 0.147–0.202) in men, against 7.0–12.3% with an average of 9.2% (52/564; CI 95%: 0.070–0.119) in women for enzymatic activity assays. However, this prevalence ranged from 14.3 to 15.4% with an estimated average of 15.1% (53/350; CI 95%: 0.115–0.193]) in men and 3.3–6.0% with an average of 4.4% (17/384; CI 95%: 0.026–0.070) in women for the genotyping studies (PCR method).

Depending on the genus and independently of the method used, the enzymatic deficiency was significantly higher in males (2.5–20.5%) compared to females (3.3–12.3%) (Table 1).

### Genetic variant involved in G-6-PD deficiency in Burkina Faso

It should be noted that the most studied deficient variant in Burkina Faso is 202A/376G G-6-PD A- variant. In the study carried out by Meissner et al. [29] in the health district of Nouna, twenty-five over thirty (25/30) of G-6-PD deficiency cases observed, were confirmed carriers of this G-6-PD A- variant. Among the 1136 samples from different genotyping studies identified, 739 samples (Table 2) were screened for mutations 376G, 202A, 542 T, 680 T and 968C with the predominance (99.8% of G-6-PD deficient cases) of 202A/376G G-6-PD A- variant.

**Table 2:** Genotypic and allelic frequencies of G-6-PD deficiency in Burkina Faso

Genotypes	n/Study	N	%
	a,b,c	Total	Total
Male			
B	99 + 41 + 54	194	55.27
A	49 + 31 + 24	104	29.63
A-	27 + 12 + 14	53	15.10
Total	175 + 84 + 92	351	100.00
Female			
BB	55 + 40 + 38	133	34.28
BA	53 + 27 + 22	102	26.29
AA	18 + 10 + 5	33	8.51
BA**-**	35 + 26 + 12	73	18.81
AA**-**	15 + 6 + 9	30	7.73
A-A-	6 + 7 + 4	17	4.38
Total	182 + 116 + 90	388	100.00

**a =** Carter et al., [27]; **b =** Ouattara et al., [14]; **c =** Ouattara et al., [28]. The data in this table come from these three references (**a**, **b** and **c**) with information allowing the calculation of the different haplotypes. The populations **a**, **b** and **c** from Ouagadougou with symptomatic or asymptomatic malaria as shown in Table 1, were conform to Hardy-Weinberg Equilibrium

$$ \mathbf{f}\ \left(\mathbf{B}\ \mathbf{haplotype}\right)=\frac{194+\left(133\ast 2\right)+102+73}{351+\left(388\ast 2\right)}=\mathbf{0.563} $$

$$ \mathbf{f}\ \left(\mathbf{A}\ \mathbf{haplotype}\right)=\frac{104+102+\left(33\ast 2\right)+30}{351+\left(388\ast 2\right)}=\mathbf{0.268} $$

$$ \mathbf{f}\ \left(\mathbf{A}\hbox{-} \mathbf{haplotype}\right)=\frac{53+73+30+\left(17\ast 2\right)}{351+\left(388\ast 2\right)}=\mathbf{0.169} $$

The study carried out by Modiano et al. [30] only screened for the 202A/376G G-6-PD A- variant whose frequency varied according to ethnic groups. In the latter study, the lowest frequency (0.069) of this variant was observed among the Fulani compared to the Mossi and the Rimaibe (0.19). However, Santamaria (376G/542T) and Betica Selma (376G/968C) variants were identified in the study carried out by Ouattara et al. [28]. G-6-PD genotypes and B, A and A- alleles frequencies are shown in Table 2.

## Discussion

It should be noted that there are very poor data on the prevalence and especially the distribution of G-6-PD deficiency genetics variants in Burkina Faso despite the context of malaria endemicity and self-medication [31–33] that would contribute to an increase in malaria mortality due to iatrogenic accidents in G-6-PD-deficient individuals. Indeed, self-medication involves risks such as maladjustment between medication and pathology, wrong dosage or drug interaction that can lead to an increase in oxidative stress.

### Prevalence of G-6-PD deficiency in Burkina Faso

The prevalence of G-6-PD deficiency was estimated to be between 9.2 and 17.0% in the various studies carried out or approximately 1,751,164 to 3,235,847 of people affected by G-6-PD deficiency in Burkina Faso. Therefore, this genetic abnormality is a public health problem requiring special attention from the country authorities in charge of health. These disparities in the prevalence could be explained by the fact that the different surveys were carried out in different parts of the country and by methodologies, which differ from one study to another.

**Fig. 2:**
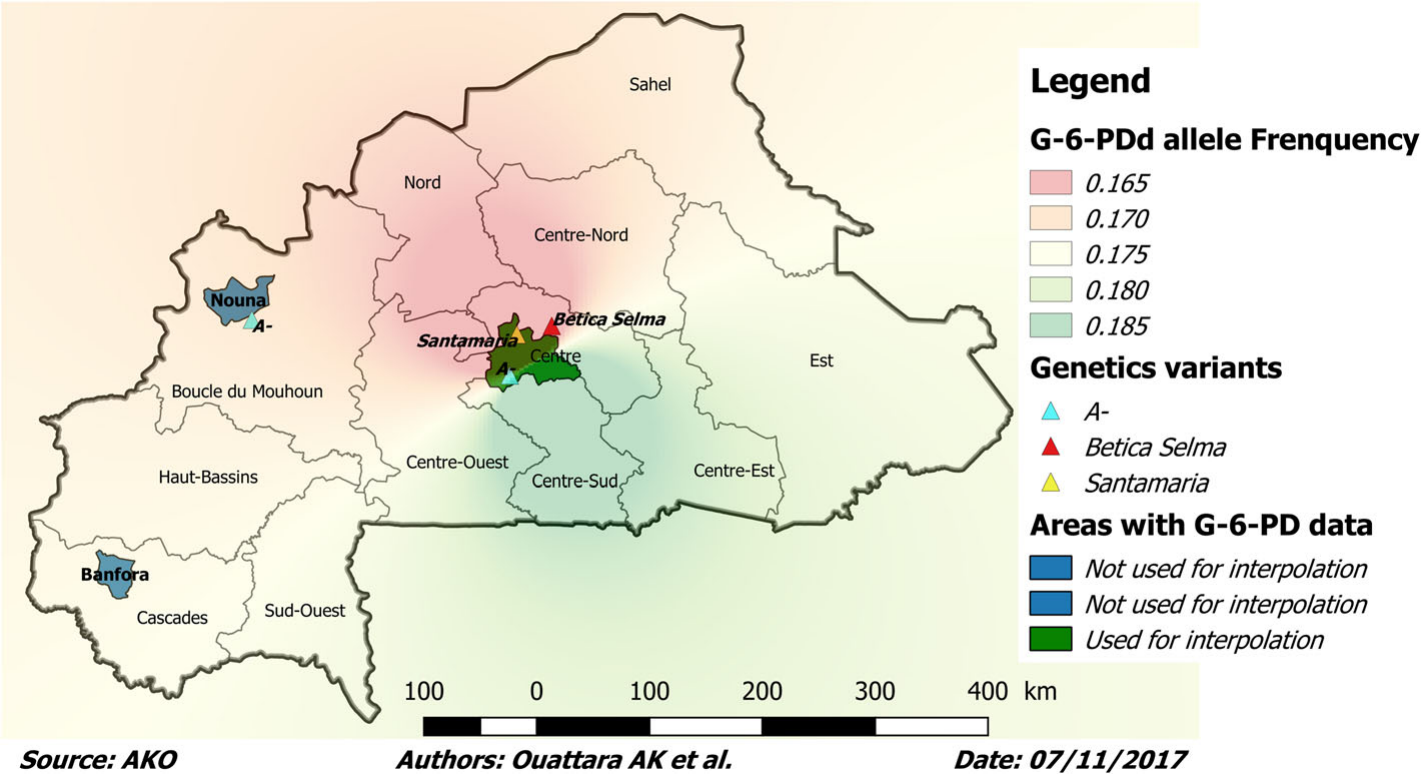
G-6-PD deficiency genetics variants and allelic frequency in Burkina Faso. The different colored areas (blue and dark green) represent the provinces or cities with data on the prevalence of the G-6-PD deficiency according to Table 1. The different genetic variants identified are represented by colored triangles (ref. [28]). Only data from dark green areas (shown in Table 2) were used for Inverse Distance weighted interpolation of the allelic frequency of G-6-PD deficiency in Burkina Faso because data from other areas did not allow the evaluation of the allelic frequency. There were no data on the G-6-PD genetics variants in Banfora because the prevalence was determined by measuring G-6-PD activity [Source: AKO]

These few studies cited with various methodologies [28, 34, 35] however, do not cover the whole national territory (Fig. 2). The prevalence of G-6-PD deficiency in Burkina Faso, therefore deserves to be determined following a national study with a standard methodology. Indeed, the variation of the methodologies in the various studies carried out greatly influences the results observed. For example, in the enzymatic studies which were the object of this systematic review, it was noted as a diagnostic technique for G-6-PD deficiency detection: the modified paper fluorescence test (NFP Test) [34], the BinaxNOW G-6-PD test [35], or spectrophotometric assay of enzymatic activity [36]. Genotyping studies are also limited by the phenotypic status of deficient heterozygous women, which are not taken into account in the prevalence of G-6-PD deficiency [14, 28].

The type of population also influences the prevalence of G-6-PD deficiency. Indeed, the frequency of this genetic abnormality will be relatively lower in groups with clinical malaria due to the mechanisms of protection against the infection progression towards a clinical form or a severe form as described by Ouattara et al. [14]. In their study, Badoum et al. [35] also showed that the prevalence of hemoglobin abnormalities and G-6-PD deficiency (6.9%) was relatively lower in children affected by *Plasmodium falciparum* symptomatic malaria. Moreover, in the different studies carried out in Burkina Faso, the highest prevalence of G-6-PD deficiency was observed among groups of people without clinical symptoms of malaria [30, 36].

### Distribution of G-6-PD gene variants in Burkina Faso

The distribution of the variants involved in G-6-PD deficiency in Burkina Faso is hard to determine. Indeed, not only the numbers of genotyping studies are poor and localized in regions, but most of all, the majority have focused on single 202A/376G G-6-PD A- variant considered as the most common in Africa [30, 34].

However, an analysis of the results of the few genotyping studies carried out in Burkina Faso and West Africa allows us to make some hypotheses. In the few studies conducted in Burkina Faso that looked for known polymorphic variants in the West African area, 202A/376G G-6-PD A- is unequivocally the most predominant variant in Burkina Faso [14, 27–29]. However, the Santamaria (376G/542T) and Betica Selma (376G/968C) variants have recently been identified Ouattara et al. [28].

In the latter study, the Santamaria variant (376G/542T) was identified particularly in an Ivorian from the Gouro ethnic group who simultaneously carried the 202A/376G variant with very low parasitemia. This demonstrates the allelic heterogeneity of the G-6-PD deficiency in West Africa and probably a relatively high frequency of the Santamaria (376G/542T) variant in Central-West Ivory Coast, mainly within the Gouro ethnic group. In view of these results, we suggest a distribution of this variant in the South-West area of Burkina Faso with a relatively higher prevalence compared to the others regions due to the community of history between certain ethnic groups (Mandingo) of this area with the Gouro (also Mandingo ethnic group) from Ivory Coast.

However, it is necessary to confirm and specify the frequency of this variant within the Gouro ethnic group in Ivory Coast. The Betica Selma (376G/968C) variant was identified in an individual of Mossi descent in the study conducted by Ouattara et al. [28]. However, we suggest a high prevalence of this variant in the northern region of Burkina Faso, mainly within the Fulani ethnic group. Indeed, the study of Modiano et al. [30] reported a low prevalence of 202A/376G variant within the Fulani ethnic group compared to the Mossi and Rimaibe despite their low susceptibility to malaria.

In addition, Maiga et al. [37] in Mali reported a high frequency of the Betica Selma (376G/968C) variant among the Fulani (6.1%) compared to the Dogon (0.0%), hence the hypothesis of a high frequency of this variant among the Fulani of Burkina Faso.

Different genotyping studies have reported three (3) G-6-PD polymorphic variants in Burkina Faso. These variants are G-6-PDB, G-6-PDA and G-6-PDA-. The present systematic review reports allelic frequencies of 0.563, 0.268 and 0.129, respectively, for the G-6-PDB, G-6-PDA and G-6-PDA- alleles in Burkina Faso. Frequencies of the last two alleles averaged around 0.39 and 0.15 respectively for the G-6-PD A allele and the G-6-PD A- allele in sub-Saharan Africa [3, 8, 18].

### Clinical manifestations of G-6-PD deficiency

The different mutations on the G-6-PD gene affect both the stability and the catalytic activity of the enzymatic protein [6, 15]. These different mutations determine haplotypes or deficient variants [38, 39], which has been classified into five (5) WHO categories according to the severity of clinical manifestations [9, 11]. Among the three deficient variants identified in Burkina Faso, the G-6-PD A- (202A/376G) and Betica Selma (376G/968C) variants have class III phenotype, while the Santamaria (376G/542T) variant has WHO class II phenotype. The class III phenotype confers moderate to mild enzyme deficiency of between 10 and 60% of the normal enzyme activity against 2-3% residual G-6-PD activity for the WHO class II phenotype [11, 26].

G-6-PD class III phenotype is associated with hemolytic anemia following oxidative stress while, the class II variants cause severe enzyme deficiency associated with acute hemolytic anemia [8, 40]. A measure of the enzymatic activity associated with these different G-6-PD variants in the context of Burkina Faso is needed for more precision on G-6-PD variants and clinical manifestation at the national level.

Indeed, the T968C allele showed a lower enzymatic activity than the other variants in a study conducted in Mexico [41]. Genetic and environmental factors may, therefore influence the clinical manifestation of G-6-PD deficiency.

G-6-PD deficiency is associated with some protection against severe *Plasmodium falciparum* malaria infections, but also with increased susceptibility to oxidant hemolysis [42, 43]. Primaquine, which in the treatment of *Plasmodium falciparum* malaria is highly effective in reducing the transmissibility of gametocytes, has concerns about its safety [43, 44]. This drug, like methylene blue, induces dose-dependent acute hemolytic anemia in individuals with G-6-PD deficiency [42–44]. Although when given at a single low dose of 0.25 mg base/kg body weight, primaquine is well tolerated regardless of the patient’s G-6-PD status, a wrong dosage through self-medication could be dangerous for G-6-PD deficient individuals [45]. Indeed, due to the absence of systematic screening, the G-6-PD status of the patient is rarely known and self-medication against malaria remains a reality for a large part of the population [31–33]. Methylene blue in combination with chloroquine for the treatment of malaria has been tested effectively in G-6-PD deficient individuals in Burkina Faso. The authors reported that this combination at a given dose was effective in fighting malaria without inducing hemolytic anemia in people with G-6-PD deficiency [34, 46, 47]. Such studies are necessary for the reduction of malaria mortality in Burkina Faso. A systematic screening of the G-6-PD deficiency and increased awareness of the use of antimalarials on medical prescriptions would therefore contribute to a considerable reduction in malaria mortality in Burkina Faso.

## Conclusion

Burkina Faso is a country where malaria is endemic with a high frequency of G-6-PD deficiency. The prevalence of this genetic disorder is estimated to average of about 15.0% in the various studies carried out. Despite the ubiquity of the 202A/376G G-6-PD A- variant in all regions of the country, it will be necessary to consider the Santamaria and Betica Selma variants whose frequencies remain to be specified in the different areas of the country. A national study with a standardized method combining genotyping and phenotyping is, therefore more than necessary to determine the actual prevalence and distribution of the different genetic variants involved. Such an approach will make it possible, on the one hand, to establish a national policy for the systematic screening of the G-6-PD deficiency, at least in the groups most at risk, namely children under five and pregnant women in order to prevent the occurrence of iatrogenic hemolytic accidents. On the other hand, it will contribute to reducing malaria mortality through the adequate management of G-6-PD-deficient individuals.

## Abbreviations

CERBA: Pietro Annigoni Biomolecular Research Center
EC: Enzyme classification
G-6-PD: Glucose-6-phosphate dehydrogenase
LABIOGNE: Laboratory of Molecular Biology and Molecular Genetics
WHO: World Health Organization
